# A case-based explainable graph neural network framework for mechanistic drug repositioning

**DOI:** 10.1093/bioinformatics/btag008

**Published:** 2026-01-14

**Authors:** Adriana Carolina Gonzalez-Cavazos, Roger Tu, Meghamala Sinha, Andrew I Su

**Affiliations:** Department of Integrative Structural and Computational Biology, The Scripps Research Institute, La Jolla, CA 92037, United States; Department of Integrative Structural and Computational Biology, The Scripps Research Institute, La Jolla, CA 92037, United States; Department of Integrative Structural and Computational Biology, The Scripps Research Institute, La Jolla, CA 92037, United States; Department of Integrative Structural and Computational Biology, The Scripps Research Institute, La Jolla, CA 92037, United States

## Abstract

Drug repositioning offers a cost-effective alternative to traditional drug development by identifying new uses for existing drugs. Recent advances leverage Graph Neural Networks (GNNs) to model complex biological data, showing promise in predicting novel drug-disease associations; however, these frameworks often lack explainability, a critical factor for validating predictions and understanding drug mechanisms. Here, we introduce Drug-Based Reasoning Explainer (DBR-X), an explainable GNN model that integrates a link-prediction module with a path-identification module to generate interpretable and faithful explanations. When benchmarked against other GNN-based link-prediction frameworks, DBR-X achieves superior performance in identifying known drug-disease associations, demonstrating higher accuracy across all evaluation metrics. The quality of DBR-X biological explanations was evaluated through multiple complementary approaches, including comparison with manually curated drug mechanisms, assessment of explanation faithfulness using deletion and insertion studies, and measurement of stability under graph perturbations. Together, these results show that DBR-X advances the state of the art in drug repositioning while providing multi-hop mechanistic explanations that can facilitate the translation of computational predictions into clinical applications.

*Availability and implementation:* DBR-X package is freely accessible from online repository https://github.com/SuLab/DBR-X

## 1 Introduction

Traditional drug discovery and development is a time-consuming and resource-intensive process (Pammolli *et al.* 2011). To address these challenges, drug repositioning has emerged as a strategic approach to reduce development costs, lower failure rates, and expedite time to market (Pushpakom *et al.* 2019). Drug repositioning strategy is based on the premise that existing drugs may exhibit effects beyond their original targets. Despite its promise, most successful drug repositioning instances are often based on clinical observations. Hypothesis-driven identification of new applications for drug candidates remains problematic due to the complexity and limited understanding of the underlying mechanisms, as well as the dispersed nature of relevant information in a growing sea of information.

A recent area in computational drug repositioning leverages knowledge graphs (KGs), which integrate heterogeneous biomedical data by representing drugs, genes, diseases, and other entities as nodes connected by typed relationships (Himmelstein *et al.* 2017, Mayers *et al.* 2022, Chandak *et al.* 2023, Gonzalez-Cavazos *et al.* 2023). By modeling biological systems as interconnected networks, KGs enable large-scale reasoning over mechanistic and functional associations that extend beyond isolated datasets. Graph Neural Networks (GNNs) further enhance this framework by learning latent representations that support accurate prediction of new therapeutic relationships (Wang *et al.* 2020, Yu *et al.* 2021, Zhao *et al.* 2021, Yang *et al.* 2022, Zhao *et al.* 2023, 2025). By aggregating information from neighboring nodes and relations, GNNs derive representations that reflect both local and broader relational context in the graph. This capability has led to strong performance in tasks including drug–target interaction prediction, disease gene prioritization, and drug repositioning. However, despite these strengths, GNN-derived representations are typically latent and difficult to interpret, making it challenging to extract explicit mechanistic rationales for predicted drug–disease associations. As a result, current KG- and GNN-based repositioning approaches often provide limited biological explanation, which constrains their practical utility for clinical translation.

To address this limitation, a growing body of work in explainable AI (XAI) has developed methods for interpreting GNN predictions. For example, GNNExplainer introduced edge and node masks to maximize mutual information with predictions (Ying *et al.* 2019), while PGExplainer used a neural model to generate explanations across instances (Luo *et al.* 2020). However, these methods may yield disconnected or incomplete subgraphs. While PaGE-Link attempted to solve this by focusing on path-based explanations using a heterogeneous path-enforcing mask, its pruning approach, which removes nodes based on their connectivity and eliminates nodes multiple hops away from the source or target, can lead to incomplete or inaccurate explanations (Zhang *et al.* 2023a).

An alternative and complementary direction draws from symbolic AI, particularly Case-Based Reasoning (CBR), which solves new problems by adapting solutions from similar prior instances (López 2013). In the context of knowledge graphs, Das *et al.* proposed a symbolic, non-parametric CBR approach for link prediction, retrieving reasoning chains from past cases to inform new predictions (Das *et al.* 2020). Their subsequent model, CBR-SUBG, extended this to a semi-parametric formulation by incorporating local subgraph similarities (Das *et al.* 2022). These methods achieve competitive link prediction performance and offer a level of interpretability through case analogy. However, they do not identify or rank specific, mechanistically relevant paths that explain why a prediction is made—limiting their use in biomedical hypothesis generation, where such paths are critical.

In this work, we introduce Drug-Based Reasoning Explainer (DBR-X), a framework that provides path-based explanations for drug-disease predictions through two complementary modules: (i) a link prediction module that identifies potential drug-disease connections by recognizing similar subgraph patterns using a CBR approach, and (ii) a path identification module that employs a heterogeneous path-enforcing mask with node degree scoring to identify mechanistically relevant connections. Unlike other GNN explainer models that identify important subgraphs without domain context, DBR-X leverages similar indication cases as templates and identifies important multi-hop paths with optimal connectivity patterns, avoiding both overly-connected hub nodes and sparsely connected nodes that may represent noise.

We trained DBR-X on a biomedical knowledge graph to predict known drug-disease candidates and provide path-based explanations. Our evaluation shows that DBR-X outperforms other GNN-link prediction frameworks as well as state-of-the-art GNN explainer baselines. The quality of top-ranked connections was assessed across multiple performance axes, including faithfulness (via deletion and insertion) and stability. Identified important connections were also validated against a external gold-standard drug mechanisms dataset. Lastly, through detailed case studies, we show how DBR-X can identify promising drug repositioning candidates for rare diseases and provide biologically plausible rationales that can guide expert evaluation and experimental validation.

## 2 Materials and methods

### 2.1 Overview of the DBR-X framework

**Figure 1:**
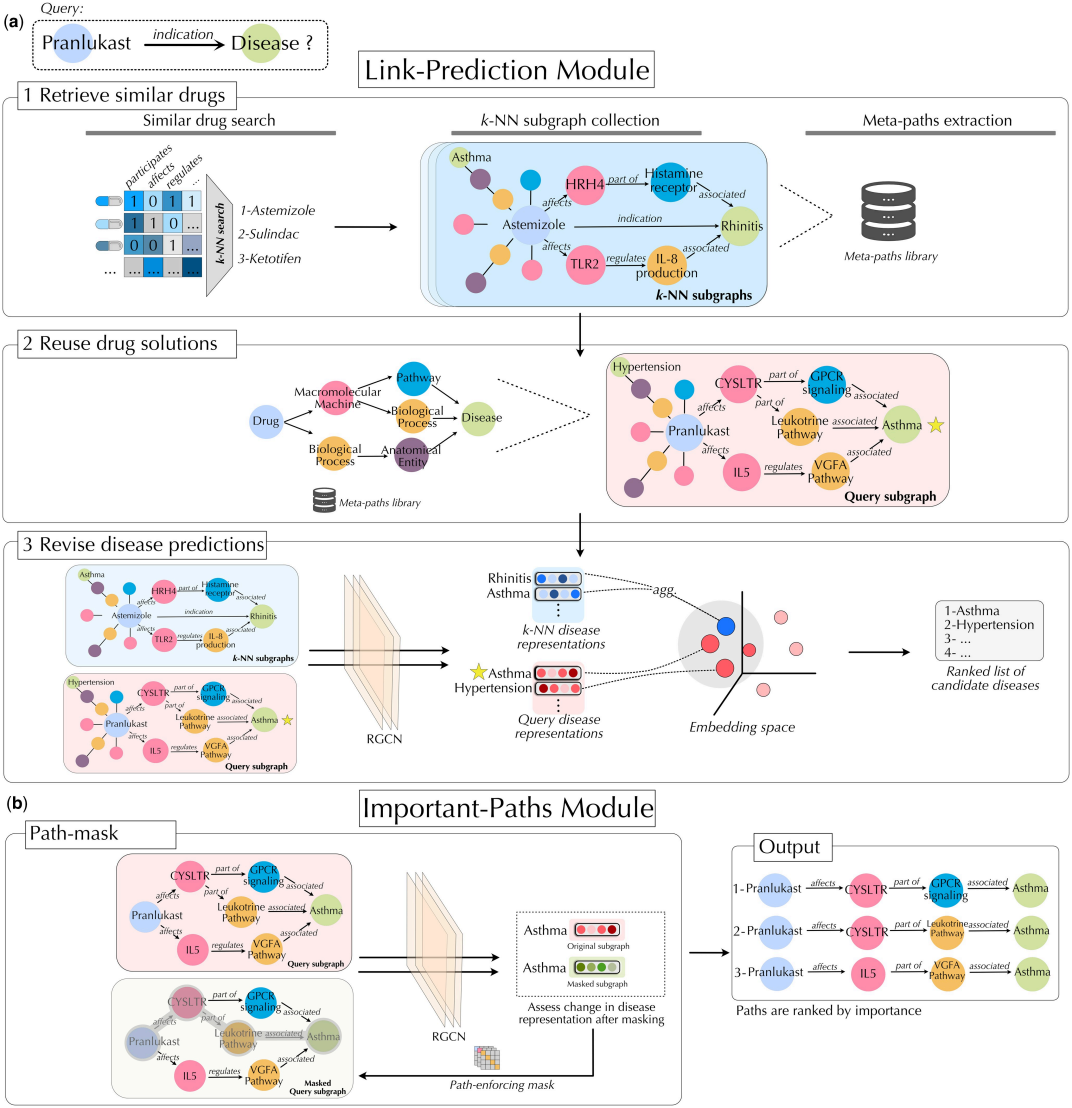
Drug repositioning predictions and explanatory paths generated by the Drug-Based Reasoning Explainer (DBR-X) framework. (a) *Link-prediction module*. (1) Given a query (Pranlukast, *indication*, ?), DBR-X retrieves *k*-NN similar drugs (Astemizole, Sulindac, Ketotifen) and collects their reasoning paths leading to known disease indications. (2) These paths are reused to construct a query-specific subgraph. (3) The most likely disease is identified by comparing disease node embeddings in the query subgraph with those from the *k*-NN drug subgraphs (highlighted with a star). (b) *Important-paths module*. A heterogeneous path-enforcing mask with node-degree scoring identifies the most informative paths supporting each prediction. The model compares disease representations between masked and original subgraphs to learn which edges are essential for preserving predictive consistency. The final output highlights the top-ranked mechanistic path explaining the Pranlukast–asthma association.

We frame drug repositioning as a link prediction task on a heterogeneous biomedical knowledge graph, where nodes represent drugs, genes, pathways, and diseases, and edges encode mechanistic biological relationships. Given a query drug *q*, the goal is to identify disease nodes *d* with plausible mechanistic support. DBR-X constructs a query-specific subgraph that focuses inference on mechanistically relevant evidence from prior drug–disease treatments and identifies the most influential edges for the prediction. DBR-X includes: (i) a case-based link prediction module that retrieves mechanistically similar drugs and transfers their reasoning patterns to the query drug, and (ii) an important-paths module that identifies the mechanistic subgraph supporting the predicted drug-disease association (Fig. 1). Full mathematical formulations and algorithmic details are provided in Supplementary S1, available as supplementary data at *Bioinformatics* online.

#### 2.1.1 Step 1: Case-Based Reasoning for drug repositioning

DBR-X follows the classical Case-Based Reasoning process of *retrieve*, *reuse*, and *revise* to generate mechanistically grounded drug–disease predictions.

1. **Retrieve similar drug cases.** Given a query drug *q*, DBR-X identifies drugs that participate in similar biological contexts within the knowledge graph. Each drug *u* is represented by a binary relation-profile vector $r_{u}\in {\left\{0,1\right\}}^{\left|R\right|}$, where each dimension corresponds to a relation type in the KG and indicates whether *u* has at least one outgoing edge of that type. Similarity between two drugs *u* and *v* is computed as cosine similarity over these profiles, $S_{uv}=\frac{r_{u}^{⊤}r_{v}}{\|r_{u}{\|}_{2}\|r_{v}{\|}_{2}}$. Drugs with higher similarity scores are interpreted as acting within comparable mechanistic environments. DBR-X retrieves the top *k* such drugs, denoted $k{-\text{NN}}_{q}$, which serve as “cases” containing prior therapeutic reasoning.
2. **Reuse mechanistic reasoning patterns.** Each retrieved drug $c\in k{-\text{NN}}_{q}$ is associated with one or more diseases it treats, connected through short mechanistic chains in the KG (e.g. drug → gene → pathway → disease). DBR-X abstracts each chain into a *meta-path*, defined as the sequence of node and relation types along the chain. It then re-instantiates these meta-paths starting from the query drug *q*, collecting all matching patterns in the KG. The union of these instantiated paths forms a query-specific subgraph $G_{i}$, which focuses attention on biologically plausible modes of action inferred by similar therapeutics.
3. **Revise candidate disease predictions.** We apply a relational graph neural network (R-GCN) to encode the query-specific subgraph $G_{i}$, producing embeddings for all disease nodes reachable from *q*. Let $d_{i}$ denote the embedding of a candidate disease in $G_{i}$, and let $D_{c}$ denote the set of diseases associated with a case drug $c\in k{-\text{NN}}_{q}$. DBR-X ranks candidate diseases by their aggregated similarity to the diseases treated by the retrieved case drugs, $\text{score}(d_{i})=\sum_{c\in k{-\text{NN}}_{q}}\text{sim}(d_{i},D_{c})$, where sim(·,·) denotes mean cosine similarity between embeddings. The top-scoring disease is returned as the predicted repositioning candidate.

Full algorithmic details, path scoring, and mask optimization are provided in Supplementary S1, available as supplementary data at *Bioinformatics* online.

#### 2.1.2 Step 2: Identifying important mechanistic paths

To explain the predicted drug–disease association, DBR-X applies a heterogeneous path-enforcing mask on the query-specific subgraph $G_{i}$. The goal is to identify the subset of edges that are most influential for the model’s prediction and that form coherent biological chains linking the drug *q* to the predicted disease *d*. The mask is defined as a set of edge-level weights $M=\{M_{e}\in [0,1]|e\in E_{i}\}$, where higher weights indicate greater contribution to the R-GCN message passing. During explanation, the pretrained GNN parameters are fixed, and only the mask is optimized. This ensures that we do not change the prediction itself, but instead uncover the minimal subgraph that supports it. DBR-X learns the mask by balancing two objectives: (i) *prediction consistency*, which encourages the masked subgraph to preserve the original disease embedding, and (ii) *path structure*, which promotes edges that form coherent mechanistic chains rather than isolated or spurious links. The overall optimization objective is:


$$L_{\text{explanation}}(M)=L_{\text{prediction}}(M)+L_{\text{path}}(M),$$

where $L_{\text{prediction}}$ measures how much the disease embedding changes when masking edges, and $L_{\text{path}}$ favors edges that participate in high-scoring mechanistic paths. Additional sparsity and entropy regularization encourages the explanation to be compact and decisive.

After optimization, the highest-scoring paths connecting *q* and *d* are extracted from the weighted subgraph using a shortest-path procedure, yielding a ranked list of mechanistic explanations.

## 3 Results

### 3.1 Case-based reasoning for drug discovery: retrieving and adapting similar drug cases

**Figure 2:**
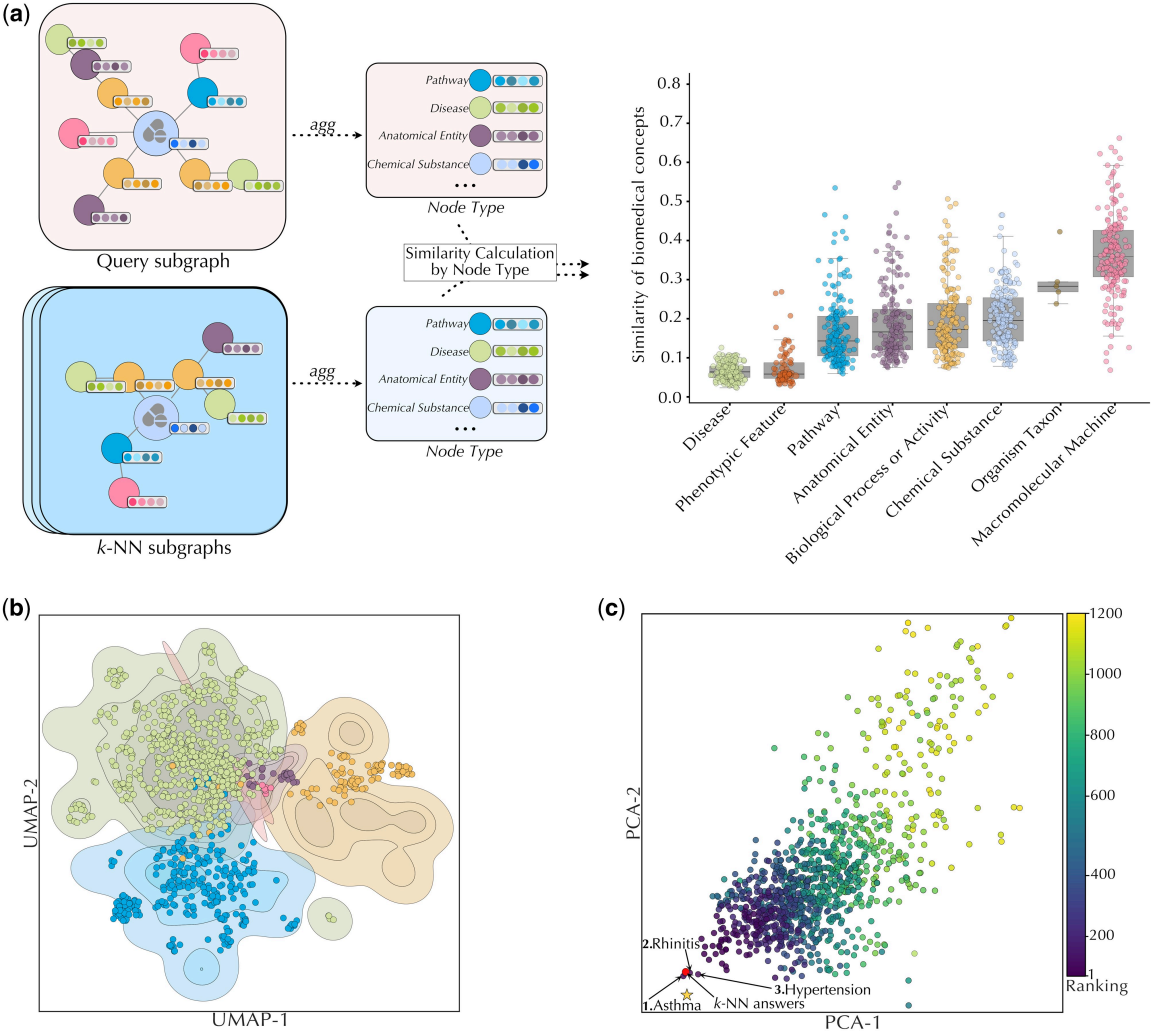
Embedding space of the DBR-X model. (a) *Structural similarity*: DBR-X preserves structural consistency between query subgraphs and retrieved drug cases. Left: example query subgraph and its corresponding *k*-NN subgraphs. For each node type, mean embeddings from the query were compared to those from similar drugs. Right: Euclidean distance distributions between corresponding entity embeddings across biological types; lower distances indicate greater similarity. (b) *Entity alignment*: UMAP projection of biomedical entities within the Pranlukast query subgraph (dots) and their counterparts from similar drugs (contour density). (c) *Disease prediction space*: PCA projection of disease embeddings for Pranlukast. The red dot marks the mean disease representation from *k*-NN drugs; the color gradient reflects repositioning ranking scores. The true known indication (star) and top-ranked predicted diseases are highlighted.

We assessed how DBR-X preserves subgraph structural similarities among similar drug cases (Fig. 2a). For this, we calculated the Euclidean distances between averaged node representations for each biological entity type (e.g. drugs, genes, pathways) within the query subgraph and the corresponding averages in the *k*-NN subgraphs. The results revealed that DBR-X consistently maintains stable representations for higher-level biological entities (pathways and biological processes), successfully capturing the shared therapeutic mechanisms connecting similar drugs. Notably, we observed higher similarity variance specifically for macromolecular machine elements—an expected pattern that reflects established pharmacological principles, where drugs with similar therapeutic effects often work through different gene targets while ultimately influencing the same biological pathways. Figure 2b then illustrates this pattern using Pranlukast as a case study, where we visualize biological entities from Pranlukast’s subgraph (shown as dots) overlaid with contour plots representing the distribution of entities from similar cases. The clear alignment between Pranlukast’s entities and the contour regions demonstrates how DBR-X successfully captures shared biological mechanisms between similar cases.

To identify potential novel repositioning candidates, DBR-X generates node representations on both the drug query subgraph and the retrieved subgraphs of similar drugs. Potential disease targets are then ranked based on how closely their representations match those of known therapeutic targets from similar drug cases. Figure 2c illustrates this process using Pranlukast as a case study. The PCA projection shows the disease prediction space, where the mean representation of diseases from similar drug cases is marked with a red dot. The visualization reveals that asthma and rhinitis, Pranlukast’s known indication, clusters near this reference point (Keam *et al.* 2003). Furthermore, the model identifies hypertension as top repositioning candidate for Pranlukast, as it also clusters close to the mean disease representation from similar cases. While no direct studies have examined Pranlukast’s effect on hypertension, it is reported that leukotriene antagonists can influence blood pressure regulation (de Silva *et al.* 2014).

### 3.2 Performance of DBR-X predictions on known indications

We evaluated DBR-X on the Mechanistic Repositioning Network with Indications (MIND) knowledge graph to assess its ability to recover known FDA-approved indications, comparing it with state-of-the-art KG completion approaches (Tu *et al.* 2024). Baselines included GNN-based link prediction models—Relational Graph Convolutional Network (R-GCN) (Schlichtkrull *et al.* 2018) and composition-based multi-relational Graph Convolutional Network (CompGCN) (Vashishth *et al.* 2019)—evaluated with ConvE (Dettmers *et al.* 2018) and DistMult (Cai and Wang 2017) scoring functions. We also compared against a non-parametric CBR baseline that matches existing path patterns without learned parameters (Das *et al.* 2020). Model performance was assessed using standard ranking metrics: Hits@*K*, measuring the frequency with which true disease indications appear among the top *K* predictions, and mean reciprocal rank (MRR), reflecting the average inverse rank of correct predictions. As summarized in Table 1, DBR-X consistently outperformed all GNN and CBR baselines across both metrics, demonstrating that focusing on query-specific subgraphs yields more accurate and mechanistically coherent predictions than whole-graph message passing.

**Table 1:** Link prediction performance.

Model^1^	MRR	Hits@1	Hits@3	Hits@5	Hits@10
RGCN + DistMult	0.1157	0.0180	0.1084	0.2108	0.3494
RGCN + ConvE	0.1197	0.0240	0.1144	0.1867	0.3614
CompGCN + DistMult	0.27735	0.1954	0.30651	0.36398	0.44828
CompGCN + ConvE	0.36112	0.25287	0.41762	0.47126	0.55556
Non-parametric CBR	0.03269	0.0158	0.0317	0.0396	0.0634
DBR-X (ours)	**0.3770**	**0.2796**	**0.4329**	**0.4789**	**0.5708**

1 Bold values denote the highest score for each evaluation metric among all models.

### 3.3 Sensitivity of multi-hop path length and neighborhood size on predictive performance

To evaluate how the number of mechanistic hops incorporated into the query-specific subgraphs affects predictive performance, we varied the maximum allowed path length (*MaxHops *= 2, 3, 4, 5) and re-trained DBR-X under each setting. As shown in Supplementary S3.1a and b, available as supplementary data at *Bioinformatics* online, performance improved markedly when increasing from 2 to 3 hops, with *MaxHops *= 3 achieving the highest overall scores (MRR = 0.3770) and consistently higher Hits@K values across multiple K thresholds. In contrast, extending the path length further to 4 or 5 hops resulted in reduced performance across metrics, likely due to the introduction of less mechanistically relevant or noisy distant connections. These results indicate that most mechanistic evidence supporting drug–disease associations is concentrated in short 2–3 hop pathways, while longer chains contribute diminishing value. Therefore, we set MaxHops = 3 as the default configuration in DBR-X.

We also evaluated the effect of the number of retrieved similar drug cases (*k*) used to construct the query-specific subgraph. As shown in Supplementary S3.1c and d, available as supplementary data at *Bioinformatics* online, retrieving only a single neighbor (k=1) resulted in poor recovery of known indications (MRR = 0.175), indicating that relying on a single case does not provide sufficient mechanistic context. Performance improved substantially when increasing to k=5 (MRR = 0.308) and continued to increase with larger neighborhoods, reaching the highest overall performance at k=15 (MRR = 0.377). Increasing further to k=20 or k=30 maintained relatively high performance but yielded diminishing or negative gains. These results suggest that DBR-X benefits from aggregating multiple mechanistically similar cases, and that moderate neighborhood sizes (k≈10–20) balance evidence diversity without introducing excessive noise. Based on this analysis, we selected k=15 as the default setting in DBR-X.

### 3.4 Identifying biologically relevant paths through path-enforcing mask learning

The second module of DBR-X generates interpretable explanations by identifying biologically meaningful pathways that link drugs to their predicted disease targets through a heterogeneous path-enforcing mask learning approach (Fig. 1b). The ultimate goal of model explanation is to enhance transparency and support decision-making. For this, we evaluated our model’s explanatory power against drug mechanisms captured by DrugMechDB, a hand-curated database of drug mechanisms (Gonzalez-Cavazos *et al.* 2023). To ensure a fair comparison, baseline models were adapted to incorporate the CBR hypothesis-driven retrieval step, yielding three variants—CBR+GNNExplainer, CBR+PGExplainer, and CBR+PaGE—each focusing on query-relevant subgraphs rather than the entire graph. Because DBR-X and CBR+PaGE-Link produce path sets, while CBR+GNNExplainer and CBR+PGExplainer output edge-level importance masks, all methods were standardized by evaluating learned masks. In this framework, edges from DrugMechDB paths were treated as positives, and all others as negatives.

**Figure 3:**
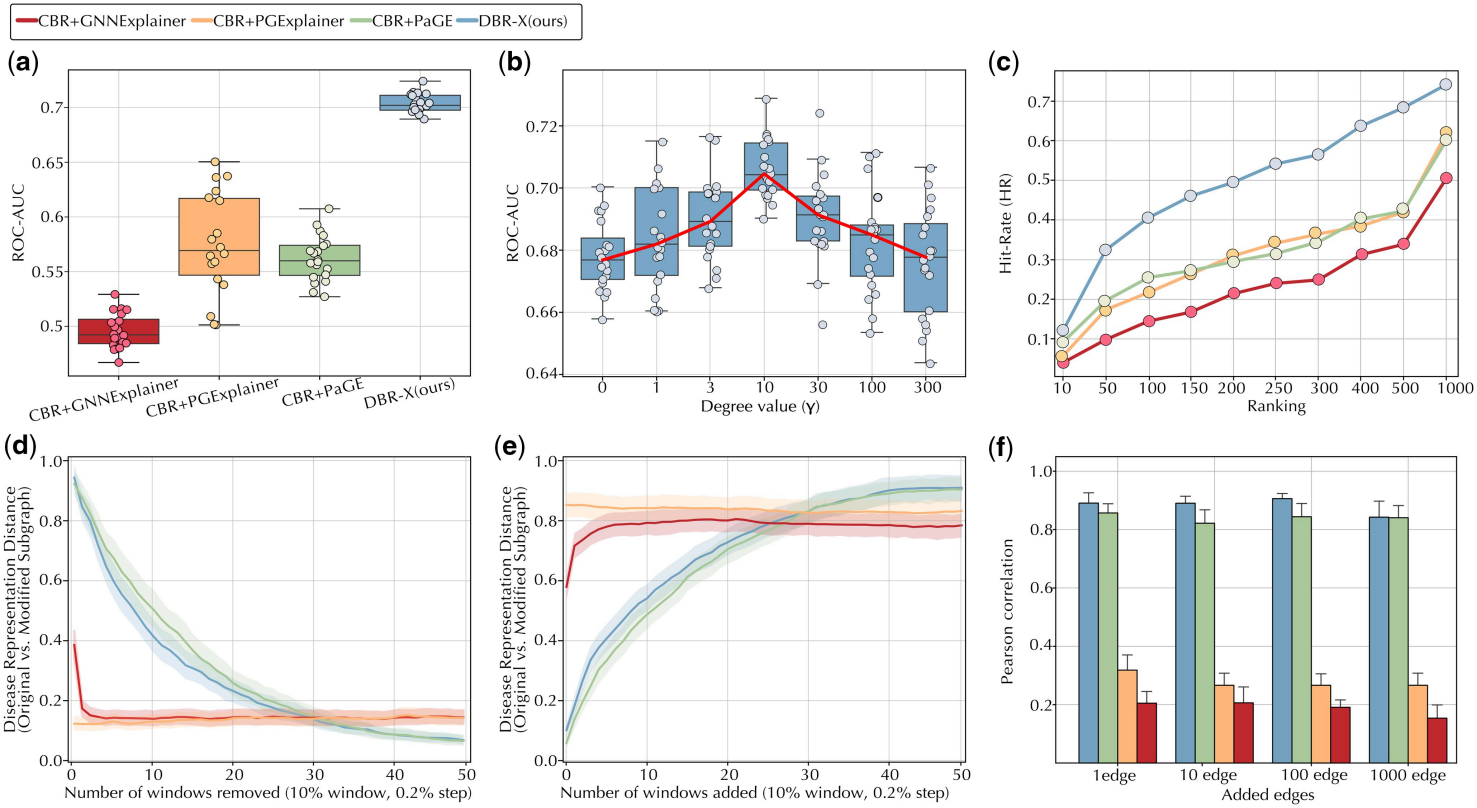
Evaluation of DBR-X important-paths module. (a) *Explanation accuracy*: ROC-AUC comparison between DBR-X and baseline models using DrugMechDB edges as ground truth; mask weights are treated as edge importance scores. (b) *Parameter sensitivity*: ROC-AUC across node degree hyperparameter (λ) values, showing its influence on explanation quality. (c) *Edge hit rate*: Proportion of ground truth edges recovered at different ranking thresholds. (d) *Deletion test*: Drop in predictive performance when high-weighted edges are progressively removed from the query subgraph. (e) *Insertion test*: Retained predictive performance when only high-weighted edges are preserved. (f) *Stability*: Robustness of explanations under random edge perturbations, measured by Pearson correlation between original and perturbed mask weights; higher values indicate greater consistency. All evaluations in (a and b) use N=20 samples. For (d and e), performance is tracked using a sliding window starting at the top 10% of edges and shifting by 0.2% steps; the first 50 windows are shown.

As shown in Fig. 3a, DBR-X achieved the highest ROC-AUC when benchmarked against DrugMechDB mechanisms. Edges from DrugMechDB were used as positive examples, and mask weights (*M*) were evaluated as edge prediction scores. A high ROC-AUC thus indicates accurate identification of mechanistic edges. The improvement stems from DBR-X’s node-degree scoring, which prioritizes paths traversing nodes with balanced connectivity (Fig. 3b). By weighting nodes with moderate degree values (λ=10), DBR-X avoids overconnected hubs and sparsely connected noise nodes, capturing pathways with clearer mechanistic relevance—an ability absent in baseline models. We also evaluated performance using the edge hit rate (HR@K), which quantifies the fraction of DrugMechDB ground truth edges recovered at different ranking thresholds. As shown in Fig. 3c, DBR-X consistently outperformed all baselines, reaching nearly double their HR values across all *K* levels and achieving full edge recovery (HR = 1) at higher ranks, whereas baseline methods plateaued at HR values between 0.35 and 0.4.

To further evaluate the biological relevance of DBR-X explanations, we compared pathway enrichment of genes extracted from the top-ranked versus bottom-ranked mechanistic paths. For each drug–disease prediction, we aggregated all genes appearing along the top-k explanatory paths and performed GO:BP, Reactome, and KEGG enrichment analysis using g:Profiler (Raudvere *et al.* 2019). Across increasing numbers of explanatory paths, top-ranked paths consistently yielded stronger and more disease-relevant enrichment signals than bottom-ranked paths (Supplementary S3.2, available as supplementary data at *Bioinformatics* online). These results demonstrate that DBR-X does not merely identify statistically important edges, but recovers mechanistically coherent biological programs that are aligned with known disease pathways.

### 3.5 Faithfulness and stability assessment of identified explanations

To evaluate the quality of explanations generated by DBR-X and ensure their reliability for drug repositioning, we assessed their faithfulness and stability using three established metrics: deletion, insertion, and stability (Lin *et al.* 1997).

The **deletion** metric evaluates the importance of edges identified by DBR-X by assessing how their removal impacts predictive accuracy. If the edges prioritized by the model’s learned mask weights are truly critical, their deletion should substantially alter the model’s ability to maintain its original predictions. To test this, we employed a sliding window approach to progressively remove edges from the query subgraph in descending order of mask weight. At each step, 10% of the edges were removed (in 0.2% increments per window), and the node representations were recomputed using the trained link prediction module. We quantified the effect of each deletion by calculating the Euclidean distance between the query disease node’s representation in the original and modified subgraphs—larger distances indicate greater disruption and thus higher edge importance. As shown in Fig. 3d, DBR-X demonstrates a sharp increase in this distance as top-ranked edges are removed, highlighting its strong explanatory faithfulness and ability to isolate a compact set of influential edges. While CBR-PaGE shows a similar performance, it shows a more gradual decrease, suggesting a less pronounced sensitivity to the removal of top-ranked edges. In contrast, CBR+GNNExplainer and CBR+PGExplainer exhibit flatter distance increases, indicating a less discerning prioritization of edges.

Complementing deletion, the **insertion** metric evaluates how well the model retains predictive accuracy when only the most important edges—ranked by mask weights—are included. This tests whether a minimal subset of high-weighted edges is sufficient to preserve the original prediction, thereby reflecting the explanatory strength of the selected connections. We started by keeping only the top 10% of edges with the highest mask weights, removing all others from the subgraph. Using the same sliding window approach (0.2% increments), edges were incrementally reintroduced in decreasing order of importance, and node representations were recomputed at each step. To quantify the effect, we calculated the Euclidean distance between the query disease node’s representation in the original subgraph and its counterpart in the modified one. If the mask effectively captures key edges, this distance should remain low when only these edges are present, indicating that they are sufficient for the model’s prediction. As shown in Fig. 3e, DBR-X displays a sharp decrease in distance when top-ranked edges are retained, confirming that a small set of highly weighted edges can reliably reconstruct the original prediction. While CBR+PaGE shows a comparable trend as DBR-X, it exhibits a slower increase, suggesting less efficiency in capturing the most critical edges. In contrast, CBR+GNNExplainer and CBR+PGExplainer display higher initial distances and flatter decreases, indicating a less discerning prioritization of edges essential for maintaining the original prediction.

Next, **stability** measures the robustness of explanations against structural perturbations, a crucial property for ensuring reliability in real-world applications where biological networks may contain noise or incomplete data. We introduced random edge additions to each query subgraph, reran the explanation algorithms, and computed the Pearson correlation between the original and perturbed mask weights. A high correlation indicates that the explanation remains consistent despite modifications. As depicted in Fig. 3f, DBR-X maintained a consistently high correlation (0˜0.85) across varying levels of perturbation, outperforming all baselines. While CBR+PaGE also showed reasonable stability (0˜0.75), DBR-X’s higher and more consistent correlation values demonstrate greater robustness of its explanations under random modifications to the query subgraph.

We further examined how the size of the retrieved neighborhood influences the concentration of learned edge weights using the same deletion and insertion procedures (Supplementary S3.3, available as supplementary data at *Bioinformatics* online). Small neighborhoods (k=1) show minimal change under deletion and limited recovery under insertion, moderate neighborhoods (k=5, k=10) show partial structure, and the sharpest deletion response and fastest insertion recovery occur at k=15. Larger neighborhoods (k=30) display more gradual deletion curves and slower reconstruction, with k=20 exhibiting intermediate behavior. Indicating that explanation concentration changes systematically with neighborhood size, as very small *k* values do not provide enough mechanistic context, while very large *k* values dilute the explanatory signal with heterogeneous evidence.

### 3.6 Validating DBR-X rationales against medical evidence

Among the approximately 7000 to 10 000 known rare diseases, only 4–6% have FDA-approved treatments, leaving the vast majority without viable therapeutic options (Fermaglich and Miller 2023). To evaluate DBR-X’s potential for addressing this significant therapeutic gap, we examined its predictions and explanatory pathways for three rare diseases that exemplify distinct therapeutic areas. We first used DBR-X’s link prediction module to identify potential repositioning candidates from the top-10 ranked predictions. We then employed the path identification module to derive the most important biological mechanism connecting each drug to its predicted disease target. Lastly, we examined their multi-hop rationales relative to existing biomedical knowledge.

**Figure 4:**
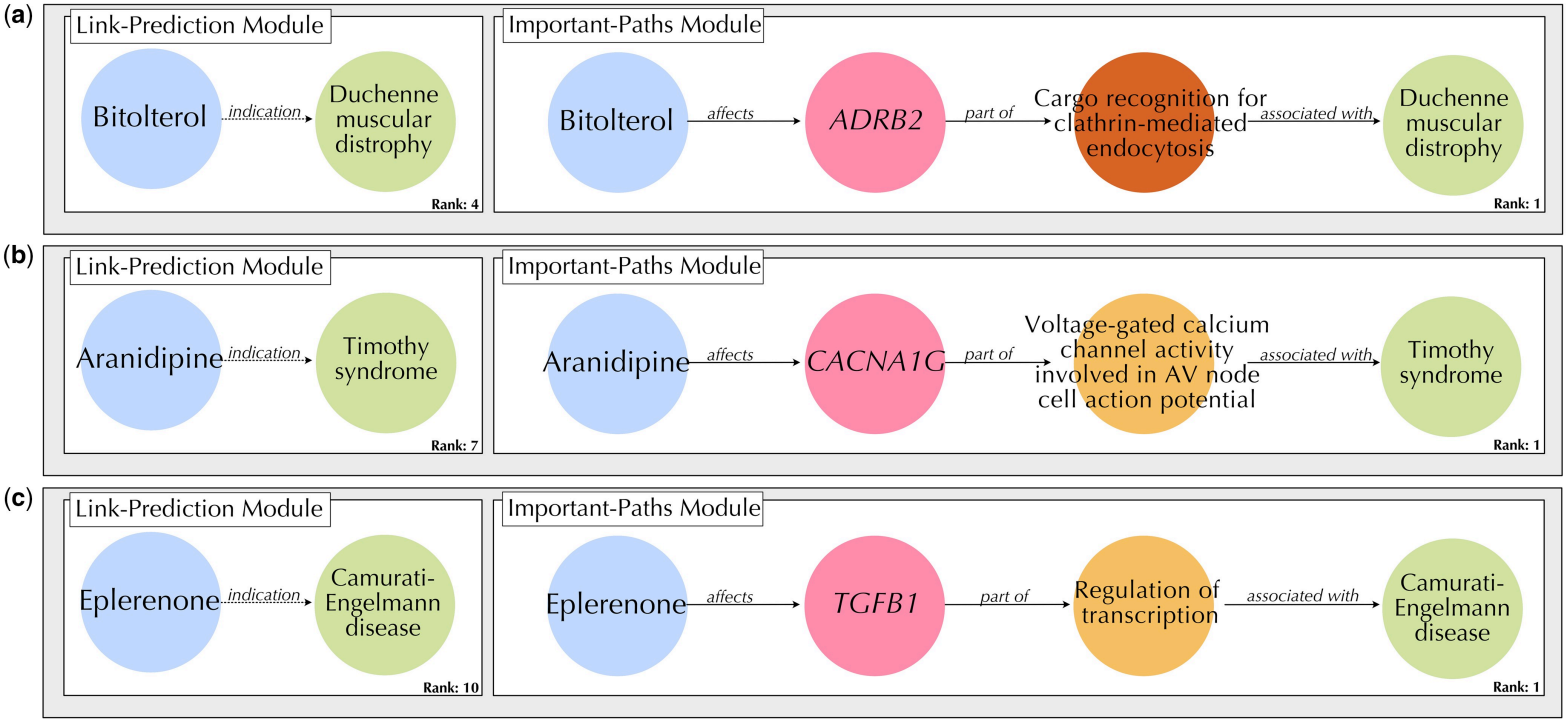
Validation of drug-repositioning candidates identified by DBR-X for three rare diseases based on medical evidence. (a) Bitolterol is predicted as a therapeutic candidate for Duchenne muscular dystrophy (rank 4), operating through *ADRB2*-mediated regulation of clathrin-mediated endocytosis. (b) Aranidipine is identified as a potential treatment for Timothy syndrome (rank 7), acting via *CACNA1G*’s role in voltage-gated calcium channel activity and AV node cell action potential. (c) Eplerenone is suggested as a therapeutic option for Camurati-Engelmann disease (rank 10), functioning through *TGFB1*-mediated transcriptional regulation.

First, we examined DBR-X prediction for Duchenne muscular dystrophy (DMD), a genetic disease characterized by progressive muscle weakness due to mutations in the dystrophin gene (Mhandire *et al.* 2022). DBR-X predicted bitolterol as a potential therapeutic agent, specifically targeting the β2-adrenergic receptor (*ADRB2*) (Fig. 4a). Bitolterol is traditionally prescribed as a bronchodilator to treat breathing difficulties, acting as an agonist of β2-adrenergic receptors. Extensive scientific literature demonstrates that β2-adrenergic receptor agonists can stimulate skeletal muscle hypertrophy by maintaining the rate of muscle protein synthesis and/or degradation (Joassard *et al.* 2013, Lynch and Ryall, 2008, Sato *et al.* 2011). The activity of these receptors is tightly controlled through clathrin-mediated endocytosis, which regulates their availability on the cell surface (Lin *et al.* 1997). This predicted mechanism suggests that bitolterol could help combat the muscle-wasting characteristic of DMD by maintaining muscle mass, potentially slowing disease progression.

We next investigated DBR-X predictions for Timothy syndrome, a rare genetic disorder affecting the heart. This condition is caused by mutations in the *CACNA1C* gene, which encodes the CaV1.2 L-type voltage-gated calcium channel, and manifests as atrioventricular block of AV cells, prolonged QT interval, and syndactyly (Tristani-Firouzi and Etheridge 2011). DBR-X identified aranidipine, an antihypertensive calcium channel blocker, as a potential therapeutic option through its interaction with the T-type calcium channel *CACNA1G* (Fig. 4b). While aranidipine typically functions by blocking both L-type and T-type calcium channels in vascular smooth muscle cells to lower blood pressure, the *CACNA1C* mutations in Timothy syndrome may reduce its effectiveness at L-type channels. Nevertheless, the drug’s ability to modulate T-type channels like *CACNA1G* suggests a promising alternative mechanism for treating the cardiac manifestations of Timothy syndrome, potentially helping to prevent atrioventricular block and normalize QT intervals.

In the final example, we looked at Camurati-Engelmann disease (CED), a rare genetic disorder affecting bone growth and development due to mutations affecting transforming growth factor-β1 (*TGFB1*). Under normal conditions, *TGFB1* maintains bone homeostasis by coordinating the activities of osteoblasts and osteoclasts. However, when mutations disrupt *TGFB1* function, patients experience progressive bone thickening, leading to chronic pain and increased fracture risk (Chen *et al.* 2022). DBR-X identified eplerenone as a promising therapeutic candidate (Fig. 4c). Eplerenone, currently used as an aldosterone receptor antagonist, has been shown to prevent atrial fibrosis by modulating the *TGFB1* signaling pathway (Du *et al.* 2017). This prediction aligns with current medical understanding, as studies in mouse models have demonstrated that targeted regulation of TGF-β1 signaling can help maintain healthy bone formation patterns (Qin *et al.* 2018).

## 4 Discussion

Understanding drug mechanisms of action remains a critical challenge in drug repositioning. Biomedical knowledge graphs integrate diverse biological relationships into structured networks, enabling systematic modeling of protein–protein interactions, drug–target bindings, and disease associations. While GNNs have shown promise in predicting novel links within these graphs, their “black box” nature limits mechanistic interpretability and clinical utility. Existing explainable GNN techniques partly address this gap but often produce explanations misaligned with biological reality. DBR-X overcomes these limitations by combining case-based subgraph construction with heterogeneous path-enforcing mask learning, leveraging the principle that similar drugs tend to act through similar biological pathways to uncover both therapeutic hypotheses and their mechanistic rationale.

DBR-X outperforms other GNN-based link prediction models by focusing on query-specific subgraphs derived from similar cases rather than performing message passing across the entire knowledge graph. While baseline approaches learn representations globally, DBR-X applies an R-GCN encoder to localized, case-relevant subgraphs, effectively filtering noise and capturing higher-order biological relationships. This case-based design offers advantages for drug repositioning: it restricts analysis to biologically meaningful pathways, reuses mechanistic patterns from successfully treated diseases, and enables inference even when direct evidence is sparse. When mechanistic information is limited, the model naturally returns smaller or less interconnected explanatory subgraphs, making the level of available support explicit.

DBR-X also provides ranked multi-hop mechanistic explanations through a path-identification module. To evaluate node contributions within these paths, DBR-X uses a degree-based weighting strategy that accounts for both hub-like and specialized biological entities. Optimal performance is observed when selecting nodes of moderate degree, which aligns with biological expectations: high-degree hubs often represent broad, non-specific processes, while very low-degree nodes may reflect noise or highly context-specific events.

DBR-X has several important limitations that should be acknowledged and addressed in future work. First, the pre-computed similarity matrix used for initial drug case retrieval relies on a simple inner product of connectivity patterns. While effective for capturing broad mechanistic contexts, this representation does not reflect multi-hop dependencies that may differentiate mechanistically similar drugs. Recent advances in attributed graph clustering couple representation learning with clustering objectives, allowing neighborhoods to form dynamically as the model learns latent structure across graph connections and attributes, rather than being fixed (Zhang *et al.* 2023b, Yang *et al.* 2025a,b). Such methods can discover overlapping and mechanistically coherent clusters, where drugs are grouped by shared multi-hop interaction motifs or functional contexts. In parallel, integrating complementary similarity modalities—such as molecular structure fingerprints and pharmacological properties—offers a natural way to enrich learned neighborhoods with orthogonal biological signals. A multimodal-adaptive approach could unify mechanistic, structural, and phenotypic dimensions within a single retrieval module, where clusters emerge from the joint evidence across knowledge-graph connectivity and external similarity sources.

In conclusion, DBR-X delivers not only novel drug repositioning predictions but also mechanistic hypotheses that can be directly tested in experimental settings. By coupling interpretability with predictive power, it bridges the gap between computational modeling and biological insight. Model interpretability plays a critical role in establishing trust in AI-assisted drug discovery and in accelerating the translation of computational findings into clinical applications.

## Supplementary Material

btag008_Supplementary_Data

## Data Availability

The MIND knowledge graph can be found at https://doi.org/10.5281/zenodo.8117748. The DrugMechDB relevant files of curated mechanism are hosted at https://doi.org/10.5281/zenodo.8139357.

## Code availability

The python code to reproduce results, documentation, and usage examples is available on GitHub at https://github.com/SuLab/DBR-X.
