# Supplementary material for: A case-based explainable graph neural network framework for mechanistic drug repositioning
Source: Bioinformatics. 2026 Jan 14;42(2):btag008. doi: 10.1093/bioinformatics/btag008 (PMC12891909; doi:10.1093/bioinformatics/btag008)
Supplement: btag008_Supplementary_Data [file btag008_supplementary_data.pdf]

S1 Model Details

Model Overview

Notation A Knowledge Graph (KG) is formally defined as a directed labeled multi-graph $G = (V, E)$, where V is a set of nodes and E is a set of edges. Facts in a KG are represented as triplets (v_1, e_1, v_2), with $v_1, v_2 \in V$ and $e_1 \in E$, where each node $v \in V$ has a type $\phi(v) \in A$ (with A being the set of possible node types) and each edge $e \in E$ has a type $\tau(e) \in R$ (with R being the set of possible edge types). The subset of edges with type $r \in R$ is denoted $E_r = \{e \in E \mid \tau(e) = r\}$. A path p in the KG is a sequence of nodes connected by edges, formally written as $p = (v_1, e_1, v_2, \ldots, e_n, v_{n+1})$, while a meta-path m abstracts this into a sequence of node and edge types, defined as $m = (\phi(v_1), \tau(e_1), \phi(v_2), \ldots, \tau(e_n), \phi(v_{n+1}))$, capturing the structural pattern of the path.

Case-Based Reasoning for drug repositioning

The link prediction module that follows the three main steps of the CBR framework; retrieving similar cases, reusing solutions, and revising predictions (Algorithm 1).

Algorithm 1 Link-Prediction Module of DBR-X

Input: Drug query q, Biomedical Knowledge Graph G,
Similarity matrix $S \in \mathbb{R}^{V \times V}$, Number of neighbors k.
Step 1: Retrieve similar drug cases
Initialize empty set k-NN$_q$
for each drug node $v \in V$ where $\phi(v) = \text{Drug}$ **do**
  if $S[q, v]$ in top-k scores **then**
    Add v to k-NN$_q$
  end if
end for
for each $c \in k$-NN$_q$ **do**
  Collect paths $P_c = \{p = (c, e_1, v_2, \ldots, e_n, d)\}$ where d is a disease node
end for
Step 2: Reuse drug solutions.
Initialize query subgraph $G_i = \langle V_i, E_i \rangle$ with $q \in V_i$
for each $c \in k$-NN$_q$ **do**
  for each path $p \in P_c$ **do**
    Extract meta-path m
    Apply m starting from q to expand G_i
  end for
end for
Step 3: Revise disease predictions.
Encode G_i and all G_j subgraphs with R-GCN (Ψ):
Set D_i as diseases in G_i
Set D_j as diseases in each G_j from c
for each disease $d_i \in D_i$ **do**
  Similarity score: $\text{sim}(d_i, D_j)$ for each G_j
end for
Optimize $\mathcal{L}_{\text{sim}}$
Return: Ranked list of D_i for q

- **Retrieve similar drug cases** Given a drug query, DBR-X first retrieves k-NN$_q$ similar drug cases c using a pre-computed similarity matrix ($S \in \mathbb{R}^{|V_{\text{drug}}| \times |V_{\text{drug}}|}$) that stores the similarity score between all pairs of drug nodes on the biomedical KG. Each drug entity u is represented as an m-hot relation profile vector $r_u \in \{0,1\}^{|R|}$, with one dimension per relation type. The tth entry $[r_u]_t = 1$ if u has at least one outgoing edge of type t, and 0 otherwise. We define the normalized vector $\hat{r}_u = r_u / \|r_u\|_2$ and compute the cosine similarity between any two drugs u and v as:

$$S_{uv} = \langle \hat{r}_u, \hat{r}_v \rangle = \frac{r_u^\top r_v}{\|r_u\|_2 \, \|r_v\|_2}.$$

The k nearest neighbors of the query q are then given by $k\text{-NN}_q = \text{TopK}_v \, S_{qv}$. For each retrieved drug case, DBR-X gathers the paths in the graph that connect the drug entity to the corresponding disease it treats. Since the number of collected paths between two nodes can grow exponentially, we uniformly sample at most $K = 1000$ paths of length up to $L = 3$ for each drug.
- **Reuse drug solutions.** For each retrieved drug $c \in k$-NN$_q$, DBR-X transfers its reasoning patterns to the query drug q through *meta-path reuse*. Each sampled path $p = (v_1, e_1, \ldots, v_{n+1})$ connecting c to a disease defines a meta-path $m(p) = (\phi(v_1), \tau(e_1), \ldots, \phi(v_{n+1}))$. DBR-X instantiates each meta-path $m(p)$ from the query node q, collecting all matching paths in the KG that follow the same sequence of node and edge types. The union of these instantiated paths forms the query-specific subgraph $G_i = (V_i, E_i)$, which aggregates the relational evidence transferred from similar drugs.
- **Revise disease predictions** Once the subgraphs of the drug query and the corresponding similar drugs are defined, DBR-X reasons across them. For this, the local subgraph structure of both the drug query and its k-NN$_q$ similar drugs is encoded with a GNN. Considering that biomedical KGs are heterogeneous graphs with labeled edges, where each edge $e \in E$ has a type $\tau(e) \in R$, we employed the multi-relational R-GCN model. We followed the general message-passing neural network scheme that iteratively updates the representation of each node by aggregating the representations of its immediate outgoing neighbors.

In particular, the general GNN message-passing process at the lth layer is given by:

$$a_v^l = \text{AGGREGATE}^l \left(\{h_s^{l-1} \mid s \in \mathcal{N}(v)\}, h_v^{l-1} \right), \quad (1)$$

$$h_v^l = \text{COMBINE}^l \left(h_v^{l-1}, a_v^l \right), \quad (2)$$

where a_v^l is the aggregated message from the neighbors, h_v^l denotes the representation of node v in the lth layer, and $\mathcal{N}(v)$ denotes the set of immediate outgoing neighbors of node v. For the multi-relational R-GCN model, which accounts for edge types, these steps are specialized as:

$$a_v^l = \sum_{r \in R} \sum_{s \in \mathcal{N}_r(v)} W_r^l h_s^{l-1}, \quad (3)$$

$$h_v^l = \text{ReLU} \left(W_{\text{self}}^l h_v^{l-1} + a_v^l \right), \quad (4)$$

where R is the set of edge types captured in the KG, $\mathcal{N}_r(v)$ denotes the immediate outgoing neighbors of node v under edge type r, derived from edges in $E_r = \{e \in E \mid \tau(e) = r\}$, W_r^l is the transformation matrix used to propagate the message in the lth layer for edge type r, and W_{self}^l is the self-loop transformation matrix that updates the node's own representation.

Candidate disease set. Let $\mathcal{D}(G_i) \subseteq V_i$ be the disease-type nodes in the query subgraph and $D_j \subseteq V_j$ the disease

nodes in each retrieved neighbor G_j. During training, DBR-X encourages the embedding of each disease node $d_i \in \mathcal{D}(G_i)$ to be more similar to the embeddings of its analogous diseases D_j than to unrelated nodes. The mean cosine similarity between normalized embeddings is

$$\mathrm{sim}(d_i, D_j) = \frac{1}{|D_j|} \sum_{d_j \in D_j} \frac{d_i^\top d_j}{\|d_i\| \|d_j\|}. \tag{5}$$

Similarity-based contrastive loss. Aggregating across all neighboring drugs retrieved yields the final contrastive objective:

$$\mathcal{L}_{\mathrm{sim}} = -\log \frac{\sum_{d_i \in \mathcal{D}(G_i)} \exp\Big(\sum_{q_j \in k\text{-NN}_{qi}} \mathrm{sim}(d_i, D_j)/\mathcal{T}\Big)}{\sum_{x_i \in \mathcal{D}(G_i)} \exp\Big(\sum_{q_j \in k\text{-NN}_{qi}} \mathrm{sim}(x_i, D_j)/\mathcal{T}\Big)}, \tag{6}$$

where $\mathcal{T} > 0$ is a temperature parameter. This loss adapts the normalized temperature-scaled cross-entropy from Chen et al. [2020] and Das et al. [2022] to align disease representations across analogous drug subgraphs.

Answer selection. After message passing, each disease node in the query subgraph obtains an embedding d_i. DBR-X ranks these nodes by their aggregated similarity to the retrieved neighbors' disease embeddings:

$$\mathrm{ans} = \arg\max_{d_i \in \mathcal{D}(G_i)} \left\{ \sum_{q_j \in k\text{-NN}_{qi}} \mathrm{sim}(d_i, D_j) \right\}, \tag{7}$$

and returns the disease with the highest score as the predicted repositioning candidate.

Heterogeneous path-enforcing mask learning

The second module of DBR-X learns a heterogeneous path-enforcing mask M to identify critical edges within the query subgraph $G_i = \langle V_i, E_i \rangle$ that explain the predicted drug-disease association (q, d) (Algorithm 2). Specifically, the heterogeneous mask is defined as $M = \{M^r\}_{r=1}^{|R|}$, where $M^r \in \mathbb{R}^{|E^r|}$ corresponds to the mask weights for edges of type r, and R is the set of edge types in the knowledge graph. To achieve this, we define the explanation graph $G_e = \langle V_e, E_e, M \rangle$ as a weighted subgraph of G_i, where $V_e \subseteq V_i$ includes the drug node q, the disease node d, and all nodes along paths connecting them, and $E_e \subseteq E_i$ consists of edges assigned weights by the mask $M = \{M_e^{\tau(e)} \mid e \in E_i\}$. Each edge $e \in E_e$ has a weight $M_e^{\tau(e)} \in [0, 1]$, reflecting its importance to the prediction, with higher weights indicating greater influence on the GNN's message-passing process. Initially, G_e is set to G_i with a random mask $M^{(0)}$, where each $M^{r(0)}$ is initialized randomly for all edge types $r \in R$, and during training, M is iteratively updated to retain edges that both preserve the predictive accuracy of the link-prediction module and form biologically meaningful paths. The goal is to refine G_e such that it captures the most explanatory subgraph connecting q to d.

Here, mask learning for link prediction explanation is done from two perspectives: important edges should be influential for the link-prediction module, and form meaningful paths. For this, we introduce the loss $\mathcal{L}_{\mathrm{explanation}}$ that allows us to achieve these two measurements:

$$\mathcal{L}_{\mathrm{explanation}}(M) = \mathcal{L}_{\mathrm{prediction}}(M) + \mathcal{L}_{\mathrm{path}}(M) \tag{8}$$

First, $\mathcal{L}_{\mathrm{prediction}}$ is the loss term for M to learn to select influential edges for model prediction. The idea is to perform a perturbation-based explanation, where parts of the input are considered important if perturbing them significantly changes the model's prediction. If removing an edge alters the original similarity between the answer node d_i of the query subgraph G_i and the disease answer node d_j of the G_j subgraphs, then the edges is a critical counterfactual edge that should be part of the explanation. This idea can be formalized as minimizing the similarity difference between the original disease answer node representation and the disease answer representation of the masked graph G_e.

$$\mathcal{L}_{\mathrm{prediction}}(M) = \sqrt{\sum_k \left([d_i]_k^{G_i} - [d_i]_k^{G_e} \right)^2} \tag{9}$$

$\mathcal{L}_{\mathrm{prediction}}$quantifies the change in the original d_i representation when it is limited to the explanation graph G_e. This way, it learns to identify candidate edges by enforcing the explanation graph to keep the original representation.

Next, $\mathcal{L}_{\mathrm{path}}$ is the loss term for M to learn to select edges that form informative paths. The mask optimization forces weights of influential edges $\sum_{e \in E_{\mathrm{path}}}$ to increase, while the mask weights of non-informative edges $\sum_{e \notin E_{\mathrm{path}}}$ to decrease. For this, we consider the weighted average of these two terms, regulated by the hyperparameters α and β.

$$\mathcal{L}_{\mathrm{path}}(M) = -\sum_{r \in R} \left(\alpha \sum_{e \in Epath} M_e^r - \beta \sum_{e \notin Epath} M_e^r \right) \tag{10}$$

To compute $\mathcal{L}_{\mathrm{path}}$ it is necessary to define the edges that form informative paths $\sum_{e \in E_{\mathrm{path}}}$. For this, we define the importance of a path with the score function $\mathrm{Score}(p)$. Each edge score is defined by the probability of including e in the explanation ($P(e) = M_e^{\tau(e)}$) and a node degree score D_s.

$$\mathrm{Score}(p) = \sum_{e \in p} \mathrm{Score}(e) \tag{11}$$

$$\mathrm{Score}(e) = log\sigma(P(e)) - D_s \tag{12}$$

We define the node degree score as $D_s = log(\kappa + |d - \gamma|)$, that measures how far a target node's degree (d) is from a specified degree score (γ). The constant κ is included to ensure that the argument of the logarithm is always positive, avoiding undefined or negative values. By applying the logarithm function, we achieve a normalized scale that mitigates the impact of large deviations and effectively handles a wide range of degree values. This scoring method provides a robust score of how closely a node's degree aligns with a defined γ score. A $\mathrm{Score}(p)$ will be high if the edges on it have high probabilities and these edges are linked to nodes with a high D_s, which would correspond to those that are closer to the defined γ. We employ Dijkstra's shortest-path algorithm to identify paths with the highest $\mathrm{Score}(p)$, selecting their edges as E_{path}. After mask learning converges, we apply Dijkstra's algorithm once more using the final mask M to generate and rank the top explanatory paths.

Step 2:

Heterogeneous path-enforcing mask learning.

The second module of DBR-X learns a heterogeneous path-enforcing mask M to identify critical edges within the query subgraph $G_i = \langle V_i, E_i \rangle$ that explain the predicted drug-disease association (q, d). Specifically, the heterogeneous mask is defined as $M = \{M^r\}_{r=1}^{|R|}$, where $M^r \in \mathbb{R}^{|E^r|}$ corresponds

to the mask weights for edges of type r, and R is the set of edge types in the knowledge graph. To achieve this, we define the explanation graph $G_e = \langle V_e, E_e, M\rangle$ as a weighted subgraph of G_i, where $V_e \subseteq V_i$ includes the drug node q, the disease node d, and all nodes along paths connecting them, and $E_e \subseteq E_i$ consists of edges assigned weights by the mask $M = \{M_e^{\tau(e)} \mid e \in E_i\}$. Each edge $e \in E_e$ has a weight $M_e^{\tau(e)} \in [0, 1]$, reflecting its importance to the prediction, with higher weights indicating greater influence on the GNN's message-passing process. Initially, G_e is set to G_i with a random mask $M^{(0)}$, where each $M^{r(0)}$ is initialized randomly for all edge types $r \in R$, and during training, M is iteratively updated to retain edges that both preserve the predictive accuracy of the link-prediction module and form biologically meaningful paths. The goal is to refine G_e such that it captures the most explanatory subgraph connecting q to d.

GNN message passing with mask. During explanation, the pretrained R-GCN parameters $\widehat{\Psi} = \{W_r^l, W_{\text{self}}^l\}_{r,l}$ are fixed, and the mask M gates message passing as:

$$a_v^l(M; \widehat{\Psi}) = \sum_{r\in R} \sum_{s\in\mathcal{N}_r(v)} M_{(s,v)}^r \, W_r^l \, h_s^{l-1}, \tag{13}$$

$$h_v^l(M; \widehat{\Psi}) = \text{ReLU}\Big(W_{\text{self}}^l h_v^{l-1} + a_v^l(M; \widehat{\Psi})\Big). \tag{14}$$

This formulation ensures that the mask modulates edge contributions while gradients are not propagated into the fixed GNN parameters $\widehat{\Psi}$.

Here, mask learning for link-prediction explanation is done from two perspectives: (1) edges should be influential for the link-prediction module, and (2) edges should form informative paths. We define the overall loss:

$$\mathcal{L}_{\text{explanation}}(M) = \mathcal{L}_{\text{prediction}}(M; \widehat{\Psi}) + \mathcal{L}_{\text{path}}(M). \tag{15}$$

Optimization setup. During the explanation stage, the pretrained GNN parameters $\widehat{\Psi}$ remain fixed, and only the mask logits Z are optimized. The mask values are obtained by applying a sigmoid transformation $M = \sigma(Z)$, which allows gradient-based updates while constraining each weight $M_e \in [0, 1]$. To guide the mask toward concise and decisive explanations, we use two regularization terms: (1) an ℓ_1 penalty (weighted by λ_1) that discourages large total mask weight and therefore pushes many edges' importance toward zero, promoting sparsity and compact subgraphs; and (2) an entropy penalty (weighted by λ_2) that penalizes uncertain mid-range values of M_e, encouraging the mask to make clear binary-like decisions between relevant and irrelevant edges. The final optimization objective is:

$$\min_Z \; \mathcal{L}_{\text{explanation}}(M(Z); \widehat{\Psi}) + \lambda_1 \sum_{e\in E_i} M_e + \lambda_2 \sum_{e\in E_i} M_e \log M_e \tag{16}$$

Here, λ_1 and λ_2 control the trade-off between explanation compactness and confidence, while $\widehat{\Psi}$ denotes the pretrained GNN weights used only for forward propagation.

Prediction-consistency loss. If removing an edge alters the original similarity between the disease answer node $d_i^{G_i} = f_{\widehat{\Psi}}(G_i; M \equiv \mathbf{1})$ and its representation under the masked graph $d_i^{G_e(M)} = f_{\widehat{\Psi}}(G_e; M)$, the edge is considered important. The loss minimizes this change:

$$\mathcal{L}_{\text{prediction}}(M; \widehat{\Psi}) = \left\| d_i^{G_i} - d_i^{G_e(M)} \right\|_2. \tag{17}$$

Path-informativeness loss. Next, $\mathcal{L}_{\text{path}}$ is the loss term for M to learn to select edges that form informative paths. The mask optimization forces weights of influential edges $\sum_{e\in E_{\text{path}}}$ to increase, while the mask weights of non-informative edges $\sum_{e\notin E_{\text{path}}}$ to decrease. For this, we consider the weighted average of these two terms, regulated by the hyperparameters α and β:

$$\mathcal{L}_{\text{path}}(M) = -\sum_{r\in R}\left(\alpha \sum_{e\in E_{\text{path}}} M_e^r - \beta \sum_{e\notin E_{\text{path}}} M_e^r\right), \tag{18}$$

To compute $\mathcal{L}_{\text{path}}$ it is necessary to define the edges that form informative paths E_{path}. For this, we define the importance of a path with the score function $\text{Score}(p)$. Each edge score is defined by the probability of including e in the explanation ($P(e) = M_e^{\tau(e)}$) and a node degree score D_s:

$$\text{Score}(p) = \sum_{e\in p} \text{Score}(e) \tag{19}$$

$$\text{Score}(e) = \log \sigma(P(e)) - D_s \tag{20}$$

The node degree score is defined as $D_s = \log(\kappa + |d - \gamma|)$, measuring how far a node's degree d is from a reference γ. The constant κ ensures positivity. Applying log normalizes deviations and mitigates large-degree effects. A high $\text{Score}(p)$ indicates paths whose edges have high inclusion probabilities and involve nodes near the target degree γ.

Path extraction. To identify the highest-scoring explanatory paths, we define an edge cost $c(e) = -\text{Score}(e)$ and apply Dijkstra's shortest-path algorithm to minimize the total cost (maximize $\text{Score}(p)$) between (q, d). The top-T shortest paths form E_{path}. After mask learning converges, Dijkstra's algorithm is run again on the final mask M to extract and rank the most explanatory paths.

Algorithm 2 Important-Paths Module of DBR-X

Input: Query subgraph $G_i = \langle V_i, E_i\rangle$, drug node q, disease node d, trained GNN model Ψ, hyperparameters α, β, γ, κ, number of epochs N

Initialize Explanation graph $G_e = \langle V_e, E_e, M\rangle$
Set epoch counter $n = 0$
while $n < N$ **do**
  Original disease representation $d_i^{|G_i|} \leftarrow \Psi(G_i, d)$
  Masked disease representation $d_i^{|G_e|} \leftarrow \Psi(G_e, d)$ using current $M^{(t-1)}$
  Compute $\text{Loss}_{\text{prediction}}(M)$
  Calculate path score $Score(p)$:
  for each edge $e \in G_e$ **do**
    Compute $Score(e)$
  end for
  Find E_{path} using edge scores $\text{Dijkstra}(G_e, q, d, \text{Score}(p))$
  Compute $\text{Loss}_{\text{path}}(M)$ according $e \in E_{\text{path}}$ and $e \notin E_{\text{path}}$
  Update $M^{(n+1)} = M^{(n)} - \eta\nabla(L_{\text{pred}}(M^{(n)}) + L_{\text{path}}(M^{(n)}))$
  $n = n + 1$
end while
Rank top explanatory paths using final mask: $\text{Dijkstra}(G_e, q, d, M^*)$
return Ranked list of explanatory paths in G_e

D Dataset

We conducted experiments on Mechanistic Repositioning Network with Indications (MIND), a biomedical knowledge graph that integrates two biomedical resources: Mechanistic Repositioning Network (MechRepoNet) [Mayers et al., 2022] and DrugCentral [Ursu et al., 2016]. Briefly, MechRepoNet is a comprehensive biomedical knowledge graph that was constructed by integrating 18 different data sources, consisting of of 9,652,116 edges, 250,035 nodes, 9 node types and 22 relations. DrugCentral, is a publicly available online resource that incorporates information from indications that have received approval from regulatory agencies. Here, knowledge graph completion prediction performance was evaluated on a subset of DrugCentral indications, for this we divided MIND into subsets: train (80%, 2087 indications), and test (20%, 390 indications).

S2. Dataset and Implementation

Hyperparameter Optimization

We performed hyperparameter tuning using Optuna [Akiba et al., 2019], running 200 trials and selecting the configuration with the highest validation MRR. The final link-prediction module consists of a two-layer Relational Graph Convolutional Network (R-GCN), where each node is initialized with a 64-dimensional feature vector and transformed into a 128-dimensional embedding. A dropout rate of 0.80 is applied during message passing to prevent overfitting. During training, we retrieve $k = 15$ mechanistically similar drug cases to construct the query-specific subgraph, and increase this to $k = 10$ at evaluation to provide additional context.

Model parameters are optimized using the Adam optimizer with a learning rate of 0.10 and weight decay of 1×10^{-5}. The contrastive sampling loss incorporates a temperature parameter $\tau = 0.1053$. We train the model using a batch size of 64 for up to 200 epochs with early stopping (patience = 20 epochs) based on validation MRR performance.

For completeness, the search space explored during tuning included:

- Learning rate: $[10^{-4}, 3 \times 10^{-1}]$
- Dropout: $[0.0, 0.9]$
- Temperature τ: $[0.01, 1.0]$
- Sampling loss weight: $[0.1, 1.0]$
- Embedding dimensionality: $\{32, 64, 128, 256\}$
- Negative sampling ratio: $\{1, 2, 4, 8\}$
- Batch size: $\{32, 64, 128\}$
- Similar drug cases train $\{5, 10, 15, 20\}$
- Similar drug cases test $\{5, 10, 15, 20\}$

Implementation details

The DBR-X model is built using the DGL [Wang, 2019] and PyTorch [Paszke et al., 2019] deep learning frameworks in Python. For data processing and computation, we utilize Pandas [McKinney et al., 2011] and NumPy [Harris et al., 2020]. Evaluation metrics are handled with scikit-learn [Pedregosa et al., 2011], while visualization is performed using seaborn [Waskom, 2021], matplotlib Hunter [2007], and UMAP [McInnes et al., 2018]. Training progress is monitored using Weights and Biases [Biewald, 2020]. The model is trained on a server equipped with a single NVIDIA Tesla V100 GPU.

Data availability

The MIND knowledge graph can be found at zenodo.8117748. The DrugMechDB relevant files for ground-truth paths are hosted at zenodo.8139357.

Code availability

The python code to reproduce results, documentation and usage examples is available on GitHub at /SuLab/DBR-X.

References

Takuya Akiba, Shotaro Sano, Toshihiko Yanase, Takeru Ohta, and Masanori Koyama. Optuna: A next-generation hyperparameter optimization framework. In *Proceedings of the 25th ACM SIGKDD international conference on knowledge discovery & data mining*, pages 2623–2631, 2019.

Lukas Biewald. Experiment tracking with weights and biases, 2020. URL https://www.wandb.com/. Software available from wandb.com.

Ting Chen, Simon Kornblith, Mohammad Norouzi, and Geoffrey Hinton. A simple framework for contrastive learning of visual representations. In *International conference on machine learning*, pages 1597–1607. PMLR, 2020.

Rajarshi Das, Ameya Godbole, Ankita Naik, Elliot Tower, Manzil Zaheer, Hannaneh Hajishirzi, Robin Jia, and Andrew McCallum. Knowledge base question answering by case-based reasoning over subgraphs. In *International conference on machine learning*, pages 4777–4793. PMLR, 2022.

Abimael González-Hernández, Jair Lozano-Cuenca, Bruno A Marichal-Cancino, Antoinette MaassenVanDenBrink, and Carlos M Villalón. Dihydroergotamine inhibits the vasodepressor sensory cgrpergic outflow by prejunctional activation of α2-adrenoceptors and 5-ht1 receptors. *The Journal of Headache and Pain*, 19(1):40, 2018.

Charles R Harris, K Jarrod Millman, Stéfan J Van Der Walt, Ralf Gommers, Pauli Virtanen, David Cournapeau, Eric Wieser, Julian Taylor, Sebastian Berg, Nathaniel J Smith, et al. Array programming with numpy. *Nature*, 585(7825): 357–362, 2020.

John D Hunter. Matplotlib: A 2d graphics environment. *Computing in science & engineering*, 9(03):90–95, 2007.

Michael Mayers, Roger Tu, Dylan Steinecke, Tong Shu Li, Núria Queralt-Rosinach, and Andrew I Su. Design and application of a knowledge network for automatic prioritization of drug mechanisms. *Bioinformatics*, 38(10): 2880–2891, 2022.

Leland McInnes, John Healy, and James Melville. Umap: Uniform manifold approximation and projection for dimension reduction. *arXiv preprint arXiv:1802.03426*, 2018.

Wes McKinney et al. pandas: a foundational python library for data analysis and statistics. *Python for high performance and scientific computing*, 14(9):1–9, 2011.

Edessa Negera, Stephen L Walker, Kidist Bobosha, Yonas Bekele, Birtukan Endale, Azeb Tarekegn, Markos Abebe, Abraham Aseffa, Hazel M Dockrell, and Diana N Lockwood. The effects of prednisolone treatment on cytokine expression in patients with erythema nodosum leprosum reactions. *Frontiers in immunology*, 9:189, 2018.

Deborah V Novack. Estrogen and bone: osteoclasts take center stage. *Cell metabolism*, 6(4):254–256, 2007.

Adam Paszke, Sam Gross, Francisco Massa, Adam Lerer, James Bradbury, Gregory Chanan, Trevor Killeen, Zeming

Lin, Natalia Gimelshein, Luca Antiga, et al. Pytorch: An imperative style, high-performance deep learning library. *Advances in neural information processing systems*, 32, 2019.

Fabian Pedregosa, Gaël Varoquaux, Alexandre Gramfort, Vincent Michel, Bertrand Thirion, Olivier Grisel, Mathieu Blondel, Peter Prettenhofer, Ron Weiss, Vincent Dubourg, et al. Scikit-learn: Machine learning in python. *the Journal of machine Learning research*, 12:2825–2830, 2011.

Barry Sloan and Noah S Scheinfeld. Pazopanib, a vegf receptor tyrosine kinase inhibitor for cancer therapy. *Current opinion in investigational drugs (London, England: 2000)*, 9(12): 1324–1335, 2008.

Oleg Ursu, Jayme Holmes, Jeffrey Knockel, Cristian G Bologa, Jeremy J Yang, Stephen L Mathias, Stuart J Nelson, and Tudor I Oprea. Drugcentral: online drug compendium. *Nucleic acids research*, page gkw993, 2016.

Minjie Yu Wang. Deep graph library: Towards efficient and scalable deep learning on graphs. In *ICLR workshop on representation learning on graphs and manifolds*, 2019.

Michael L Waskom. Seaborn: statistical data visualization. *Journal of Open Source Software*, 6(60):3021, 2021.

S3. Supplemental Figures

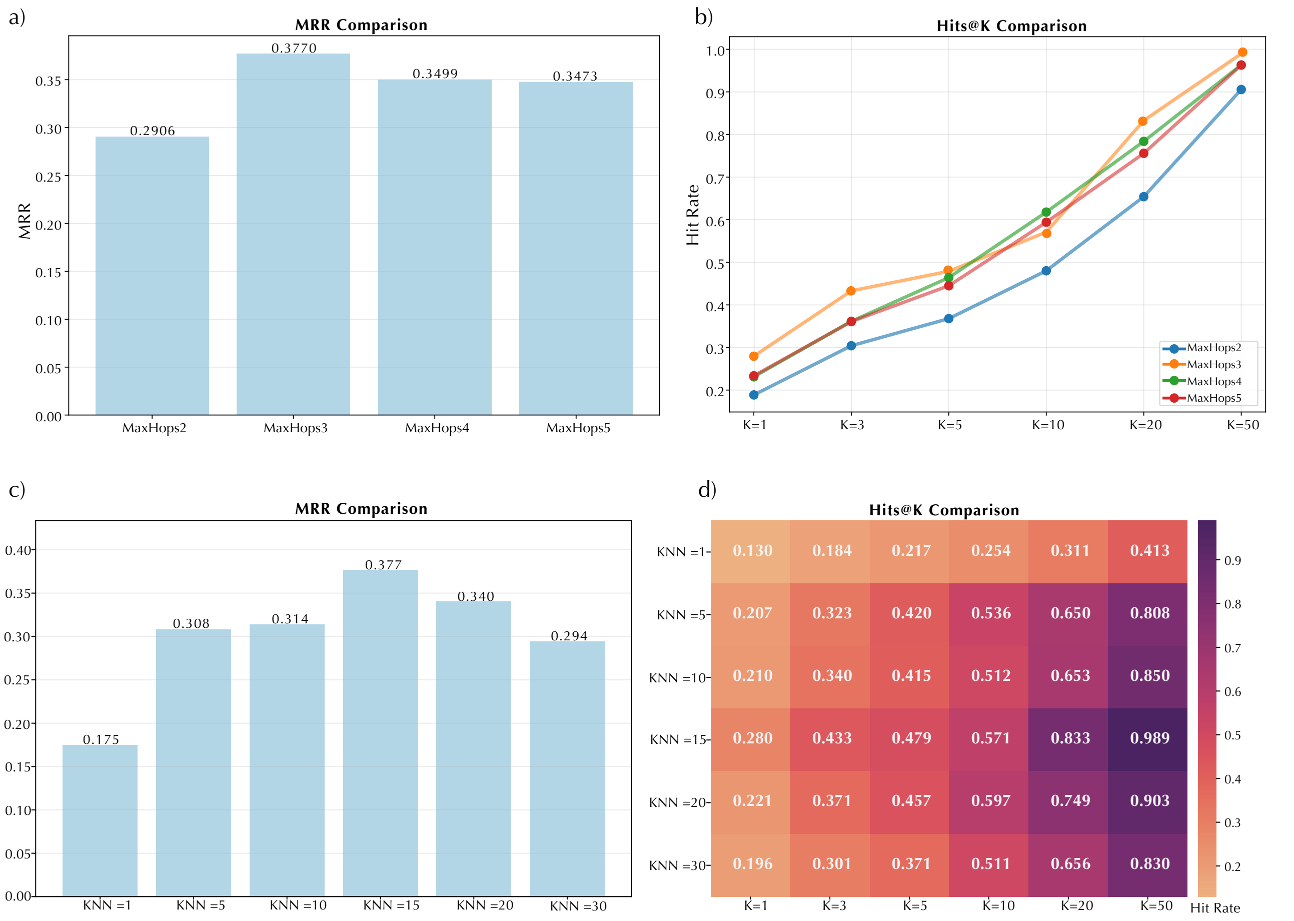

Fig. 1. Sensitivity of DBR-X predictive performance to mechanistic path length and neighborhood size.(a) Mean reciprocal rank (MRR) across models trained using different maximum allowed path lengths ($MaxHops$ = 2, 3, 4, 5). Performance increases when moving from 2 to 3 hops, with $MaxHops = 3$ achieving the highest accuracy, while longer paths (4-5 hops) lead to reduced performance. (b) Corresponding Hits@K comparison (K = 1, 3, 5, 10, 20, 50), showing consistent improvement up to 3 hops and declines beyond that depth. (c) MRR performance across different numbers of retrieved similar drug neighbors (k = 1, 5, 10, 15, 20, 30). Accuracy improves substantially as k increases, peaking at $k = 15$. (d) Heatmap of Hits@K across neighborhood sizes, illustrating that moderate neighborhood sizes ($k \approx 10$–20) yield the strongest overall performance. Together, these results show that DBR-X benefits from compact multi-hop mechanistic context and moderate case-based neighborhood sizes, supporting the selection of $MaxHops = 3$ and $k = 15$ as default settings.

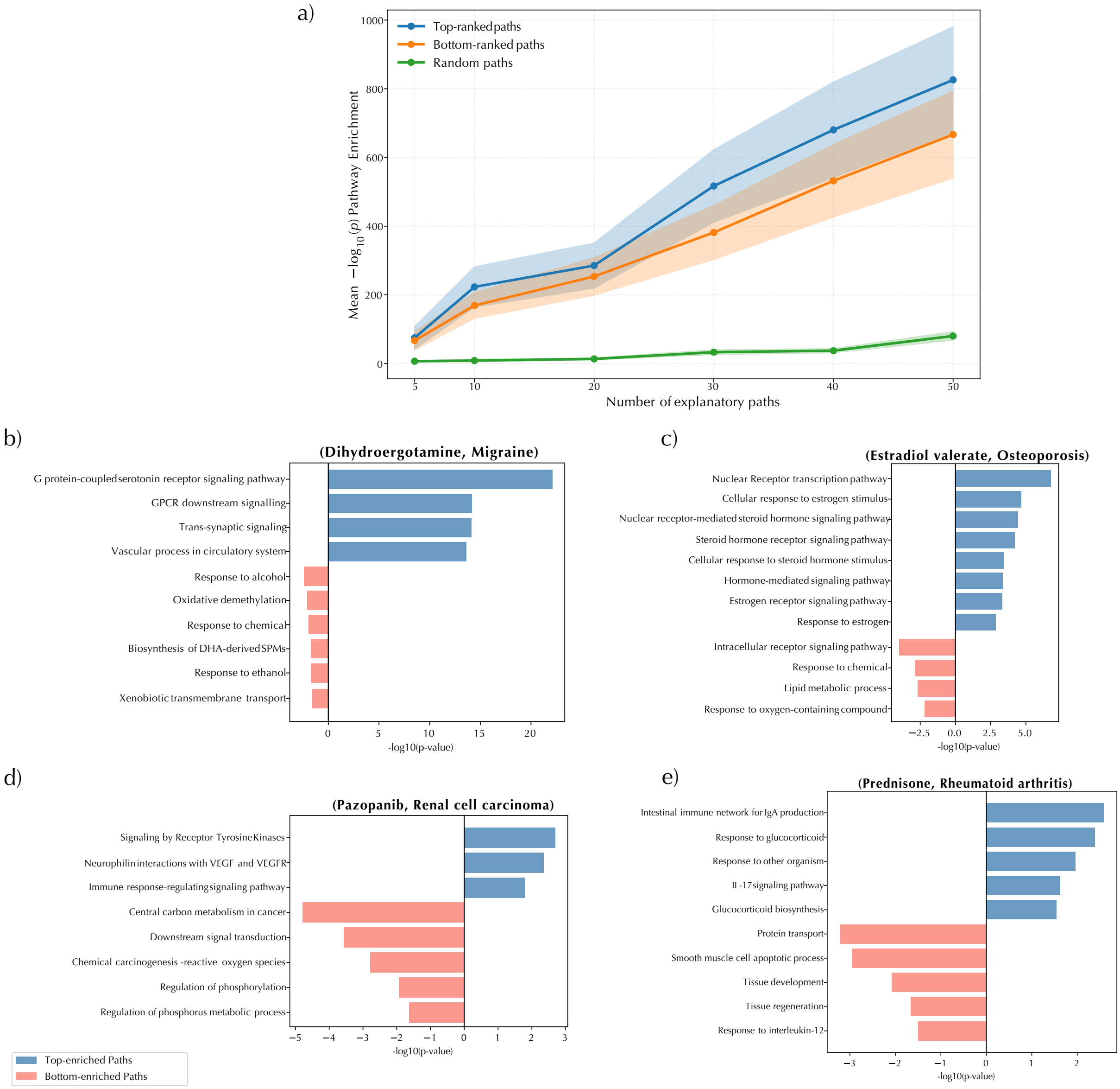

Fig. 2. Enrichment of genes from top- versus bottom-ranked mechanistic paths. (a) For each drug–disease prediction, we compared pathway enrichment (GO:BP, Reactome, KEGG) of genes extracted from the top-k and bottom-k ranked explanatory paths. Top-ranked paths consistently showed stronger enrichment than bottom-ranked paths. (b) *Dihydroergotamine → Migraine.* Top-ranked paths were enriched for G protein–coupled serotonin receptor signaling, GPCR downstream signaling, and trans-synaptic neurotransmission—consistent with dihydroergotamine's modulation of 5-$HT_{1B/1D}$, receptors to constrict blood vessels in the head González-Hernández et al. [2018]. Bottom-ranked paths were enriched for generic xenobiotic and small-molecule metabolic processes lacking migraine-specific mechanistic relevance. (c) *Estradiol valerate → Osteoporosis.* Top-ranked paths captured estrogen receptor–mediated transcriptional signaling that regulates the balance between osteoclast and osteoblast activity Novack [2007]. In contrast, the bottom-ranked paths were enriched for general chemical response and lipid metabolic processes, which lack direct relevance to bone remodeling. (d) *Pazopanib → Renal cell carcinoma.* Top-ranked mechanistic paths were strongly enriched for VEGF/VEGFR receptor tyrosine kinase signaling and Neuropilin-mediated angiogenic co-receptor interactions, consistent with Pazopanib's role in inhibiting angiogenesis through VEGFR blockade Sloan and Scheinfeld [2008]. The bottom-ranked paths predominantly involved generic cancer-associated metabolic reprogramming and oxidative stress responses. (e) *Prednisone → Rheumatoid arthritis.* Top-ranked explanatory paths were enriched for glucocorticoid receptor–mediated immunoregulation, including response to glucocorticoids, IL-17 signaling, and IgA immune network pathways, reflecting Prednisone's suppression of Th17-driven synovial inflammation and autoantibody-producing B-cell responses in RA Negera et al. [2018]. In contrast, bottom-ranked paths were enriched for nonspecific processes (e.g., protein transport, tissue development, smooth muscle apoptotic process), which do not reflect the targeted immunosuppressive mechanism

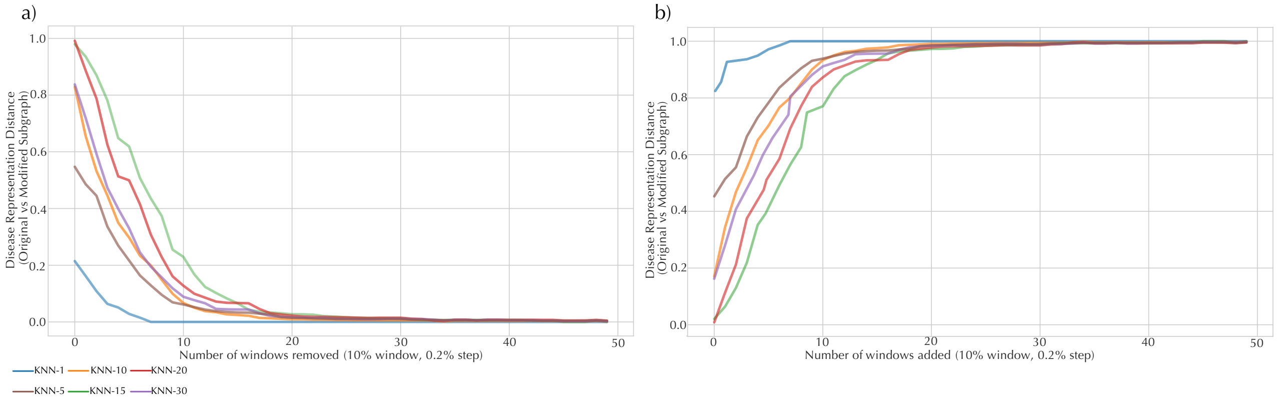

Fig. 3. Deletion and insertion analyses across neighborhood sizes. (a) Deletion test: Disease representation distance when progressively removing top-ranked edges (10% window, 0.2% step). Minimal change for $k = 1$, moderate change for $k = 5$, a pronounced disruption at $k = 15$, and more gradual curves for $k = 10$ and $k = 30$, with $k = 20$ showing intermediate behavior. **(b)** Insertion test: Representation distance when only the highest-weighted edges are retained and additional edges are added back (10% window, 0.2% step). Recovery is slow for $k = 1$ and $k = 5$, fastest at $k = 15$, and increasingly gradual again for $k = 10$ and $k = 30$, with $k = 20$ recovering at an intermediate rate.
